# Hail netting excludes key insect pests and protects from fruit damage in a commercial Minnesota apple orchard

**DOI:** 10.1093/jee/toad197

**Published:** 2023-10-31

**Authors:** Sally G A Nelson, Annie E Klodd, William D Hutchison

**Affiliations:** Department of Entomology, University of Minnesota, 1980 Folwell Avenue, St. Paul, MN 55108, USA; University of Minnesota Extension, 1420 Eckles Avenue, St. Paul, MN 55108, USA; Department of Entomology, University of Minnesota, 1980 Folwell Avenue, St. Paul, MN 55108, USA

**Keywords:** exclusion netting, IPM, physical control, fruit quality

## Abstract

Exclusion netting in some European and North American apple (*Malus domestica* Borkhausen, Rosaceae, Rosales) orchards has been documented to be an effective method of control for multiple insect pest species. By minimizing reliance on insecticides, these orchards have reduced costs, risks to the environment and non-target species, and reduced the risk of insecticide resistance. This study examined the use of commercially available hail netting (DrapeNet®; Prosser, WA) as a pest exclusion strategy under conditions in Minnesota, USA. In 2021 and 2022, we assessed the efficacy of hail netting as a tool for pest suppression in orchards by monitoring pest species in netted and open plots crossed with and without insecticide applications. Our findings show that both of the major pest species in Minnesota, the codling moth (*Cydia pomonella* L.; Lepidoptera: Tortricidae) and the apple maggot (*Rhagoletis pomonella* Walsh; Diptera: Tephritidae), were significantly reduced inside the netting compared to open plots by 94% and 96%, respectively. For a secondary pest, the red-banded leafroller (*Argyrotaenia velutinana* Walker; Lepidoptera: Tortricidae), moth populations were reduced by 56%. We also found that insecticide application alone did not significantly reduce pest pressure in these species. Additionally, we investigated the subsequent effects of hail netting on fruit quality and yield. The use of hail netting and insecticide application resulted in significantly higher proportions of high-quality fruit at harvest. However, netting did not significantly influence yield. These findings suggest that hail netting can be used to control Midwest apple insect pests with limited insecticide applications while maintaining high fruit quality.

## Introduction

Conventional orchards that produce apples (*Malus domestica* Borkhausen, Rosaceae, Rosales) are highly managed agricultural ecosystems that heavily rely on pesticides to minimize pest-related losses to fruit quality or yield. A 2021 National Agriculture Statistics Service survey found that 85% of US apple acreage was treated with insecticide (USDA 2022). Growers prioritize risk mitigation practices due to consumer preference for pristine fruit, weather events, and variability in annual yield (Menapace et al. 2013). This risk aversion often results in repeated application of insecticides to control the complex of insect pests, of which the most notable in Minnesota are the codling moth (*Cydia pomonella* L.; Lepidoptera: Tortricidae), and the apple maggot (*Rhagoletis pomonella* Walsh; Diptera: Tephritidae) (McCamant et al. 2007, Sisson 2009). A study in Quebec conservatively estimated that pesticide-free plots can suffer 22 and 42% fruit damage from codling moth and apple maggot, respectively (Chouinard et al. 2016). Following a conventional calendar-based spray schedule, insecticide applications for apple maggot have historically been applied every 10–14 days, averaging 5–6 applications per year (Gadoury et al. 1989). Spraying frequency has decreased with the adoption of integrated pest management (IPM) methods, but both codling moth and apple maggot continue to warrant multiple sprays each year in most orchards (Kadoić Balaško et al. 2020). Mating disruption has been a key technology for the sustainable management of codling moth in large production areas in North America such as those in the Pacific Northwest and Northeast United States (Thomson et al. 2001, Brunner et al. 2005, McGhee et al. 2011). At this time, mating disruption and other non pesticide-based control tactics developed for the codling moth in major apple production areas in North America are not always feasible or are not well-studied in the small and noncontinuous orchards of the Midwest (Witzgall et al. 2008). Consequently, growers in the Midwest are in need of sustainable IPM strategies that are effective against multiple insect pest species regardless of orchard size or spatial continuity.

The use of netting has gained popularity in pome fruit orchards worldwide, with several different designs to protect from inclement weather, sunburn, and insect pests (Chouinard et al. 2016). The codling moth's development of resistance to various classes of insecticides and biologicals necessitated the adoption of alternative strategies (Sauphanor et al. 2000, Kelderer et al. 2010, Knight 2010). One strategy was the development of a single-row exclusion netting system called Alt’Carpo (2.2 × 5.4 mm mesh) in France (Romet et al. 2010). Over the last 20 yr, the suppression effect of exclusion netting used in pear (*Pyrus* sp.) and apple orchards on codling moth reproductive behavior, and their subsequent ability to infest fruit, has been thoroughly documented in French and Italian orchards (Alaphilippe et al. 2016). In Italy, it has also been documented that flat hail netting suspended horizontally over trees can interfere with male codling moth's ability to approach and mate with females, resulting in reduced fruit damage (Tasin et al. 2008). Another Italian study in apple showed that single-row hail netting (2.4 × 4.8 mm mesh) effectively excluded a pest complex composed of Tortricid moths, *Halyomorpha halys* Stål (Hemiptera: Pentatomidae), and *Drosophila suzukii* Matsumura (Diptera: Drosophilidae), with no detrimental effect on fruit quality at harvest time (Candian et al. 2018).

In North America, there have been several studies documenting the effectiveness of different types of exclusion netting for insect pests in apple (Chouinard et al. 2016). In Quebec, Canada, a 5-yr study documented the effectiveness of a single row, complete exclusion netting system (1.9 × 0.95 mm mesh) that prevented damage from the apple maggot, codling moth, and the tarnished plant bug (*Lygus lineolaris* Palisot de Beauvois, Hemiptera: Miridae) (Chouinard et al. 2017, 2022). More recently, a study in Washington found that codling moth could be excluded by using net cages (2 × 5 mm mesh) placed around large orchard blocks (Marshall and Beers 2021, 2022). While successful at reducing fruit injury initially, these cages did allow a few individuals through, and over time moths were able to establish a population within these blocks (Marshall and Beers, 2023). Additionally, the investigators found that while the cages effectively excluded large, flying insects, smaller and less mobile insects such as aphids were consistently more abundant inside cages than outside (Marshall and Beers 2021, 2022). Leafrollers, such as the oblique-banded (*Choristoneura rosaceana* Harris; Lepidoptera: Tortricidae), can also establish higher population numbers under netting (Chouinard et al. 2022). Both the apple maggot and codling moth are able to move through mesh sizes that are similar to those employed in hail netting (variable mesh size: 7.4 × 3 mm, 6 × 1.8 mm, etc.) (Sauphanor et al. 2012, Chouinard et al. 2022, Marshall and Beers 2023). It is believed that effective exclusion is due to both small mesh sizes and interference with mate-seeking behavior in the codling moth (Tasin et al. 2008, Marshall and Beers 2023).

In Europe, Australia, North America, and South America, studies have shown that netting can create a microclimate effect, protecting fruit from sunburn, hail, wind, and other factors with minor or no significant effects on fruit quality (Bosco et al. 2015, Mupambi et al. 2019). For example, netting can buffer temperature and light exposure on covered trees, protecting fruit while still allowing photosynthesis (Chouinard et al. 2019). While netting can mitigate adverse conditions throughout the season, overall, fruit quality is mostly unaffected by the presence of netting in apple orchards (Chouinard et al. 2019, Candian et al. 2020). A review of protective netting studies in apple found that most often, changes in fruit quality were driven by environmental conditions, rather than the presence of netting (Mupambi et al. 2019). The most common change in fruit under netting was delays in fruit maturity and color development (Chouinard et al. 2019, Mupambi et al. 2019).

In Minnesota, hail netting has primarily been adopted for its ability to protect fruit from hail damage. Whether or not such netting can also be used to exclude insect pests, protect fruit, and ultimately reduce insecticide use, has not previously been investigated in the Midwest. In this study, we explored the efficacy of commercially available drape-style hail netting as a pest exclusion tactic in comparison with insecticide use. In 2021 and 2022, we assessed the efficacy of hail netting by comparing the presence of 3 pest species in netted and open plots in production systems with the presence/absence of insecticide application. These pest species include the codling moth, apple maggot, and red-banded leafroller (*Argyrotaenia velutinana* Walker, Lepidoptera: Tortricidae). We chose to monitor the red-banded leafroller as it is slightly smaller than the oblique-banded leafroller, easy to monitor with a pheromone trap, and is known to have moderate to high populations in Minnesota with 3 generations per year (Fadamiro 2004). In addition to pest exclusion, we investigated the subsequent effects of hail netting on fruit quality and yield. We also assessed the influence of netting on light intensity and temperature by monitoring these conditions in the open and under netting.

## Materials and Methods

### Experimental Design

**Fig. 1.**
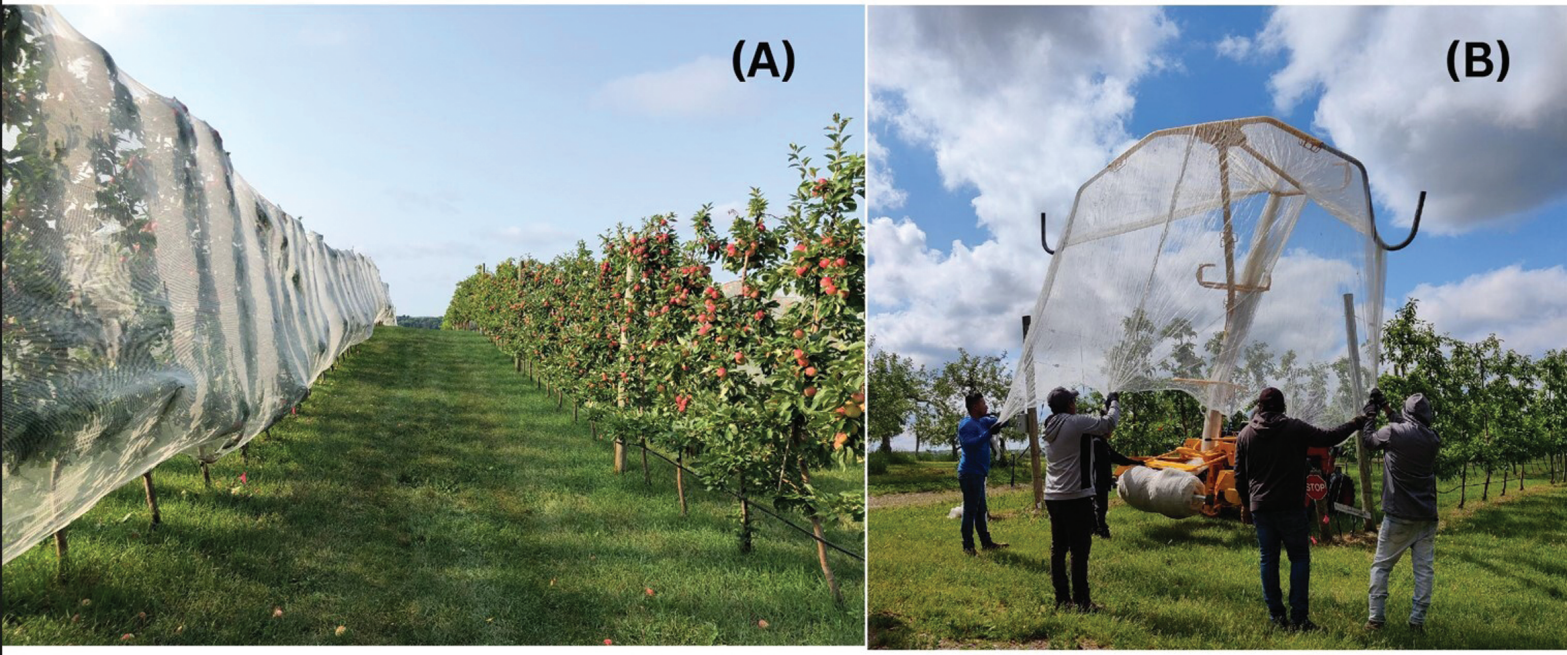
Photos showing (A) Drape Net hail netting on a high-density trellis system and (B) the NetWizz applicator used to deploy the netting over a single row of trees. Photos taken by Sally Nelson.

During 2021 and 2022, field trials were conducted at 2 commercial apple orchards to determine the effect of hail netting on insect pest abundance, apple marketability, and yield. Orchards were located in White Bear Lake (Washington Co.; 45.108182, -92.950315) and Preston (Fillmore Co.; 43.682833, -92.074867), Minnesota. The studies were conducted on high-density trellis plantings of SweeTango apple, where all trees were mature (planted in 2015 in White Bear Lake and 2009 in Preston), and fruit-bearing age at the time of this study. Netting used in our study was Drape Net white monofilament netting (mesh size, 6 × 1.8 mm, DrapeNet® North America, Prosser, WA) and was 12 meters wide (Fig. 1A).

**Fig. 2.**
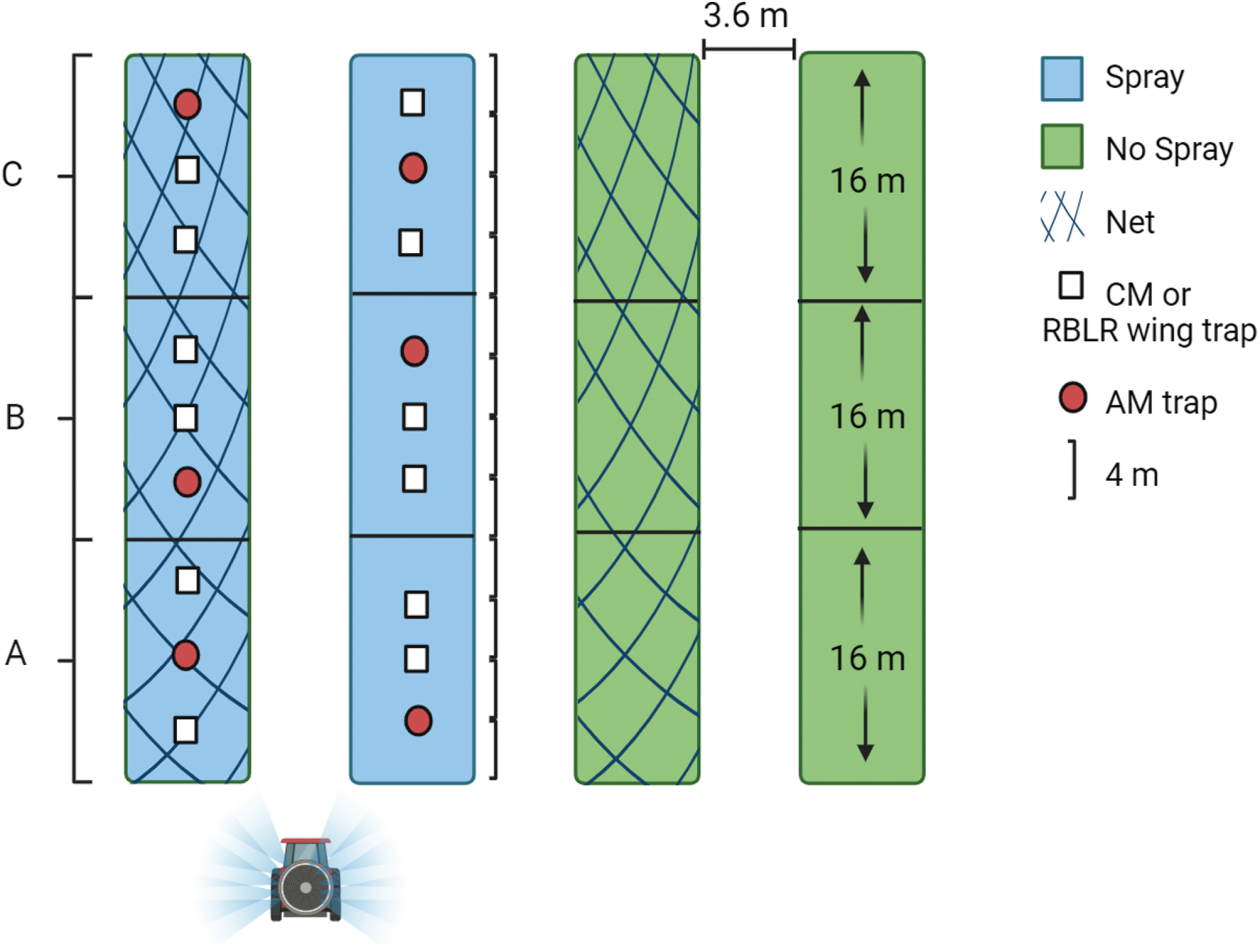
Illustration of an experimental block. Four adjacent rows were used for the 2 × 2 factorial design, with each row representing a different treatment. Shown are sample layouts of insect traps with spacing between traps. Spacing between plots within the block are also shown. Each plot was divided into three subsections (A, B, C) in 2021 or 2 subsections (A, B) in 2022, with one trap for each species placed into each subsection. Spacing of subsections of each plot are shown.

Four adjacent rows of SweeTango apple were used for each experimental block (replicate), with each block using independent rows (Fig. 2). Each experimental block was approximately 48 m (2021) or 32 m (2022) long and 15 m wide, with an average spacing of ~1.3 m between individual trees within a row and ~3.6 m between rows. The study design required that selected rows of trees be left uncovered by netting and untreated with insecticide, for use as an untreated check treatment. Due to the economic risk that untreated rows and potential fruit loss posed to the grower, we were limited to 2 experimental blocks, 1 at each location, in 2021. The experiment was expanded in 2022 to 5 experimental blocks, with 2 experimental replicates at White Bear Lake and 3 at Preston. In total, we had 7 total replicates across both years, with 3 in White Bear Lake and 4 in Preston. Experimental block placement was dependent on the location of SweeTango apples in each of the orchards, so the distance between blocks was variable. In 2022, the 2 experimental blocks at White Bear Lake were ~350 m apart; and the 3 experimental blocks at Preston were each separated by 3 buffer rows (~12 m).

A 2 × 2 factorial experimental design was implemented to compare the effect of netting to that of the grower’s current IPM program, which included trap-based monitoring and insecticide use (Fig. 2). The design consisted of 4 treatments per experimental block: each treatment represented by 1 row within each block. Treatments were factorial combinations of netting (either present or absent) and insecticide application (“spray” and “no spray”), and are referred to as Net & Spray, Net & No Spray, No Net & Spray, and No Net & No Spray (Fig. 2). The spray treatment received insecticide application following the grower’s IPM program, and the no spray treatment was not sprayed with any insecticide after the netting was applied (Table 1).In the spray treatment, 5 insecticide applications took place in both 2021 and 2022 at each orchard (Table 2). The first spray each year coincided with petal fall and targeted plum curculio (*Conotrachelus nenuphar* Herbst; Coleoptera: Curculionidae), and the European red mite (*Panonychus ulmi* Koch; Trombidiformes: Tetranychidae), neither of which were subjects of this study due to their early-season presence prior to the application of hail netting. None of the insecticide treatments applied in either year targeted the red-banded leafroller, as there is no action threshold established for this species as it poses minimal risk in Minnesota.

**Table 1.** Dates of netting application, trap deployment and removal, and fruit harvest for White Bear Lake and Preston, MN, 2021 and 2022.

Year	Location	Date	Event
2021	White Bear Lake	26 May	Netting application
		2 June	Traps deployed
		1 Sept	Traps removed
		1 Sept	Harvest
	Preston	1 June	Netting application
		3 June	Traps deployed
		31 Aug	Traps removed
		31 Aug	Harvest
2022	White Bear Lake	6 June	Netting application
		7 June	Traps deployed
		2 Sept	Traps removed
		2 Sept	Harvest
	Preston	8 June	Netting application
		9 June	Traps deployed
		30 Aug	Traps removed
		31 Aug	Harvest

**Table 2.** Insecticide applications shown for the orchard in White Bear Lake, MN. The orchard in Preston, MN was sprayed using the same succession of sprays and using the same products, with dates varying by up to 7 days. All insecticide applications were timed based on insect monitoring, 2021–2022.

Year	Date	Insecticide	Trade Name	EPA Reg. No.	Rate (kg/ha)	Target Pest
2021	May 18	clothianidin	Belay	59639-150	0.42	*C. nenuphar*
	May 18	abamectin	Agri-Mek 0.15 EC	100-898	0.28	*P. ulmi*
	June 8	novaluron	Rimon 0.83 EC	66222-35	2.80	*C. pomonella*
	July 5	acetamiprid	Assail 30 SG	8033-36	0.56	*R. pomonella*
	Aug 1	acetamiprid	Assail 30 SG	8033-36	0.56	*R. pomonella*
2022	June 1	clothianidin	Belay	59639-150	0.42	*C. nenuphar*
	June 1	abamectin	Agri-Mek 0.15 EC	100-898	0.28	*P. ulmi*
	June 17	novaluron	Rimon 0.83 EC	66222-35	2.80	*C. pomonella*
	July 13	acetamiprid	Assail 30 SG	8033-36	0.56	*R. pomonella*
	Aug 4	acetamiprid	Assail 30 SG	8033-36	0.56	*R. pomonella*

Insecticide application rates in Imperial units: Belay – 6 oz./acre, Agri-Mek – 4 oz./acre, Rimon – 40 oz./acre, Assail – 8 oz./acre.

### Netting Application and Removal

The grower installed the hail netting using standard procedures described below. Hail netting was applied to the tree rows after petal fall, ensuring that the pollination of apple blossoms was complete before application. Dates for netting application are provided in Table 1. The net applicator, NetWizz (DrapeNet® USA, Prosser, WA), was attached to the back of a slow-moving tractor (Fig. 1B). The NetWizz suspends the netting over the top of a row, allowing the netting to unroll and drape over a single row of trees. This process was facilitated by 3–4 orchard employees ensuring even coverage over the tree canopy. The 2 edges of the netting were zip-tied (DrapeNet® USA, Prosser, WA) on the underside of the tree canopy, near the base of the trees every 3–4 m to hold the net in place. As a result, the netting forms a covering around all of the branches on the trees, with only the trunks excluded from the netting. There was incomplete closure of the netting on the underside of the tree canopy, at the gaps between zip-ties, at a height ranging from 0.5 to 1 m. The netting remained on the trees from petal fall until harvest, covering the trees throughout fruit development (Table 1). Netting was removed from the rows using the NetWizz as anticipated harvest dates approached.

### Insect Monitoring

Following the application of hail netting, insect traps for 3 pest species were deployed across all treatments and experimental blocks at both locations (Table 1). Traps were placed ~4 m apart in both 2021 and 2022. Each row was divided evenly lengthwise into 2 (2022) or 3 (2021) subsample sections for trap placement (Fig. 2). A trap for each of the 3 insect species was placed within each of these subsample sections. In 2021, 3 traps for each of the 3 insect species, nine traps total, were placed within each row in each experimental block. In 2022, subsample C was dropped, so that 2 traps for each of the 3 insect species, 6 traps total, were placed within each row in each experimental block (Fig. 2). Traps were randomly assigned a position (first, second, or third) within each subsample group (Fig. 2). Traps were set 1.5–2 m off the ground in the tree canopy, with at least 4 m between traps. In the event of tree gaps within the row, traps were placed further apart, exceeding a 4 m separation. In netted rows, traps were placed under the netting in the tree canopy.

Trécé PHEROCON (Trécé Inc., Adair, OK) wing-style traps (plastic top-GL/TR-3302, paper liner-GL/TR-3323) were baited with species-specific sex pheromone lures (Great Lakes IPM, Vestaburg, MI) and used to trap both codling moth (lure-GL/TR-3111) and red-banded leaf roller (lure-GL/TR-3103). Red ball traps (GL/GL-1040) were used to monitor apple maggot, baited with an apple essence lure (GL/GL-3039) and covered in TAD All-Weather adhesive coating (previously sold as Tanglefoot Sticky Coating, GL/TA-1565; Great Lakes IPM, Vestaburg, MI). For both years, trap contents were counted and removed weekly. Dates for deployment of traps were delayed slightly in 2022 due to later bloom and netting application (Table 1). Sex pheromone lures for codling moth and red-banded leaf roller were replaced every 3 wk, with unused lures being stored in a freezer (−17.5 °C). The sticky liners in the Trécé PHEROCON traps were also replaced every 3 wk. The foliage around the red ball traps was removed following manufacturer guidelines so that 30 cm of space around the trap was open. Apple essence lures were placed greater than 30 cm away from the red ball traps and these lures were not replaced during the season per recommendations from the manufacturer.

### Apple Quality

At harvest, samples of fruit from within each experimental row were evaluated and rated following SweeTango club variety specifications. Apples were selected at random, but evenly throughout various canopy heights in trees within each subsample section (Fig. 2) in each experimental row. These subsample sections were the same divisions of each plot that were used for the insect trap placement (A, B, and C) (Fig. 2). Twenty-four apples were taken from each subsample section in both years, for a total of 1,350 apples evaluated for marketability between years. Fruit was rated on the basis of several measures including skin color (% red), size, deformity, russeting, and blemishes (Supplementary Table 1). The fruit was rated as either Extra Fancy (EF), Fancy (F), Minneiska (M), Utility (U), or Cull (C) based on SweeTango club variety standards. Extra fancy and fancy apples are those that can be acceptably sold as the SweeTango variety in the fresh market. Minneiska apples are also sold fresh market but are considered to be second-tier quality due to superficial blemishes. Utility apples are those that are used in processing, such as for baked goods, apple sauce, and cider. Cull apples are not able to be used for any purpose.

To determine skin color, the percent of the apple’s skin that was red was determined by visual approximation. As this is a subjective measure, several reference apples were used to depict variation in color to maintain consistency in approximation. For size, a caliper (Cranston Machinery Co., Oak Grove, OR) was used to measure the diameter of the fruit at its widest point. For blemishes, an open wound was defined as a wound with broken skin, rotted skin, or visible flesh (examples: bird peck, insect tunneling). A closed blemish was defined as a blemish where the skin of the fruit is not punctured but there is evidence of damage (examples: sunburn spot, bruise, oviposition scar). For the purposes of fruit rating, closed blemishes were also divided into small and large categories: small being less than or equal to 9.5 mm, and large being greater than 9.5 mm (3/4 in.) at their largest point. Any misshapenness resulting in asymmetrical apples was noted as deformed. For russeting, the percent of the apple skin exhibiting russeting was recorded using visual approximation. Russeting is a natural feature of the SweeTango apple and is not considered a blemish, however, large and/or solid blotches can affect the marketability. A subset of the apples harvested for marketability was also used to evaluate soluble solids (Brix), a measure of ripeness in fruit crops. We used one apple from each subsection (A, B, C) from our marketability sample, resulting in 3 (2021) or 2 (2022) apples per experimental plot being used for this measure. Combined over 2 yr, we had 64 apples in this analysis. Brix represents the amount of soluble sugar in the fruit juice as a percentage, so a relatively higher Brix percentage correlates to higher sugar content and riper fruit. To evaluate soluble solids, a drop of juice was placed on a portable refractometer (Shen Zhen YIERYI Technology Co., Ltd. Shen Zhen City, Guang Cong Province, China). Apples were individually blended to ensure brix evaluation was from juice throughout the apple, not from a single slice (Harker et al. 2002).

### Apple Yield

At harvest, a section of each experimental row was chosen at random to be harvested for yield estimates. Apples were hand-picked and weighed in the field using a portable field scale (Doran Scales, Inc.; Batavia, IL). In 2021, three, 4 m (~12 ft) sections were randomly selected from within each of our experimental rows, and all apples within that section were picked and weighed. Therefore, 12 m/row of apples were picked in each experimental row in 2021. This method created some challenges: if a tree was on the edge of the 4 m section, only part of the apples on the tree would be picked. Thus, spotters were needed to ensure apples were picked consistently throughout the canopy. Therefore, in 2022, 4 trees were randomly selected from each experimental row and all apples from those trees were picked and weighed. These high-density trees are planted every 1.3 m (4 ft) allowing 2 trees to be converted to 2.6 m/row. Therefore, 5.2 m/row of apples picked in each experimental row in 2022. This methodology was more efficient and easier to communicate to crew members harvesting the apples. In 2022, 1 of the experimental blocks at Preston was harvested before we could collect our samples, resulting in 6 blocks for apple quality data and yield between the 2 yr.

### Temperature, Light Intensity, and PAR Measures

Temperature and light intensity sensors (HOBO Pendant MX Temperature/Light Data Logger; Onset Computer Co., Bourne, MA) were deployed inside a netted and an un-netted row in both orchard locations in both years. Sensors were hung by twist-ties from a branch on the inner canopy approximately 2 m (6 ft) from the ground. In 2021, HOBO sensors were deployed on 30 June in White Bear Lake and 6 July in Preston. In 2022, HOBO sensors were deployed on the same day as the trap installation (Table 1). Photosynthetic active radiation (PAR; Xtreem, Dataflow Systems Ltd. Environmental Monitoring; Christchurch, New Zealand) loggers were taped (Gorilla Tape, ULINE, Hudson, WI) to a steel t-post (Chicago Heights, IL) attached to the trellis wire within a row. The PAR sensors were clear of any tape or obstruction and were approximately 1.3 m (4 ft) from the ground. One sensor was installed under netting and another in an open row in White Bear Lake in both years. The PAR loggers were installed on 21 July 2021; and on 7 June 2022 (the same day as the insect trap installation). All sensors were removed on the same day as insect trap removal in both years and in both locations (Table 1). Daily light integral (DLI) results are based on data from PAR loggers, which were consistent with daily light intensity measures taken by the HOBO data loggers. DLI was calculated using the average PAR logged per day as a function of light intensity and duration (Faust and Logan 2018).

### Statistical Analysis

Insect monitoring data were analyzed using R (version 4.2.2) in RStudio (R Core Team 2022, R Studio 2020). A separate analysis was conducted for each of the insect pest species. For analyses of the number of insects trapped per week, we used analysis of variance (ANOVA) in a 2 × 2 factorial design in a linear mixed effects model (package: nlme, functions: lme, anova; Pinheiro et al. 2023). Trap catches from different subsections within each experimental block were included as separate responses and multiple measurements were accounted for in the random effects term. Fixed effects included terms for netting (yes/no), spray (yes/no), and their interaction, with random effects of experimental block nested within week. We used a square root transformation (sqrt(*y* + 0.5)) for the response variable, the number of insects trapped per week, to satisfy the assumptions of the model. Graphical inspection of residual plots indicated that the analytical assumptions of the model were met for all analyses.

For harvest marketability, analyses were conducted using logistic regression in a generalized linear mixed effects model framework with a logit link function (packages: nlme, MASS, lmerTest; functions: glmmPQL, summary; Venables and Ripley 2002, Kuznetsova et al. 2017, Pinheiro et al. 2023). Fixed effects included terms for net, spray, and their interaction as previously stated, with a term for block as the random effect. In order to analyze this data using binomial logistic regression, we condensed our rating scale into a binary variable in 3 separate analyses. In the first analysis, EF and F ratings were combined while M, U, and C ratings were combined. This analysis was used to determine if fixed effects influenced the proportion of apples that were of the highest quality and able to be sold as SweeTango variety, which are the most valuable fresh market apples to consumers. This group will be referred to as 1st quality apples. In the second analysis, EF, F, and M were combined to represent all apples that are considered to be of fresh market quality, with U and C making up the second group. This analysis was used to determine if our treatments influenced the proportion of apples that were able to be sold as fresh market apples. In the third analysis, EF, F, M, and U apples were combined into 1 group to represent apples that are marketable, as fresh or for processing, versus those that are a total loss (C).

Individual marketability parameters as response variables were analyzed using either generalized linear mixed effect models or linear mixed effects models (package: nlme, function: lme, Pinheiro et al. 2023). A generalized linear mixed effect model was used for binary variables of skin color (>60% red/not), russeting (>25% coverage/not), blemishes (yes/no), and deformity (yes/no); and linear mixed effects models were used for fruit size (mm) and soluble solids (Brix). All models used terms for spray, net, and their interaction as fixed effects, and random effects of block nested within year.

All yield data were analyzed using ANOVA within a linear mixed effects model framework (package: nlme, Pinheiro et al. 2023). Fixed effects included terms for net, spray, and their interaction, with random effects terms of experimental block nested within year. Graphical inspection of residual plots indicated that analytical assumptions were met for all analyses.

Daily average temperature (DAT) was calculated from temperatures logged every 15 min. To determine if temperatures under the netting were different from those outside, ANOVA within a linear mixed-effects model was used to analyze DAT data, with a term for net as the fixed effect and a term for logger as the random effect. Graphical inspection of residual plots showed that analytical assumptions of a linear mixed effects model were met.

## Results

### Insect Exclusion

#### Codling moth.

**Fig. 3.**
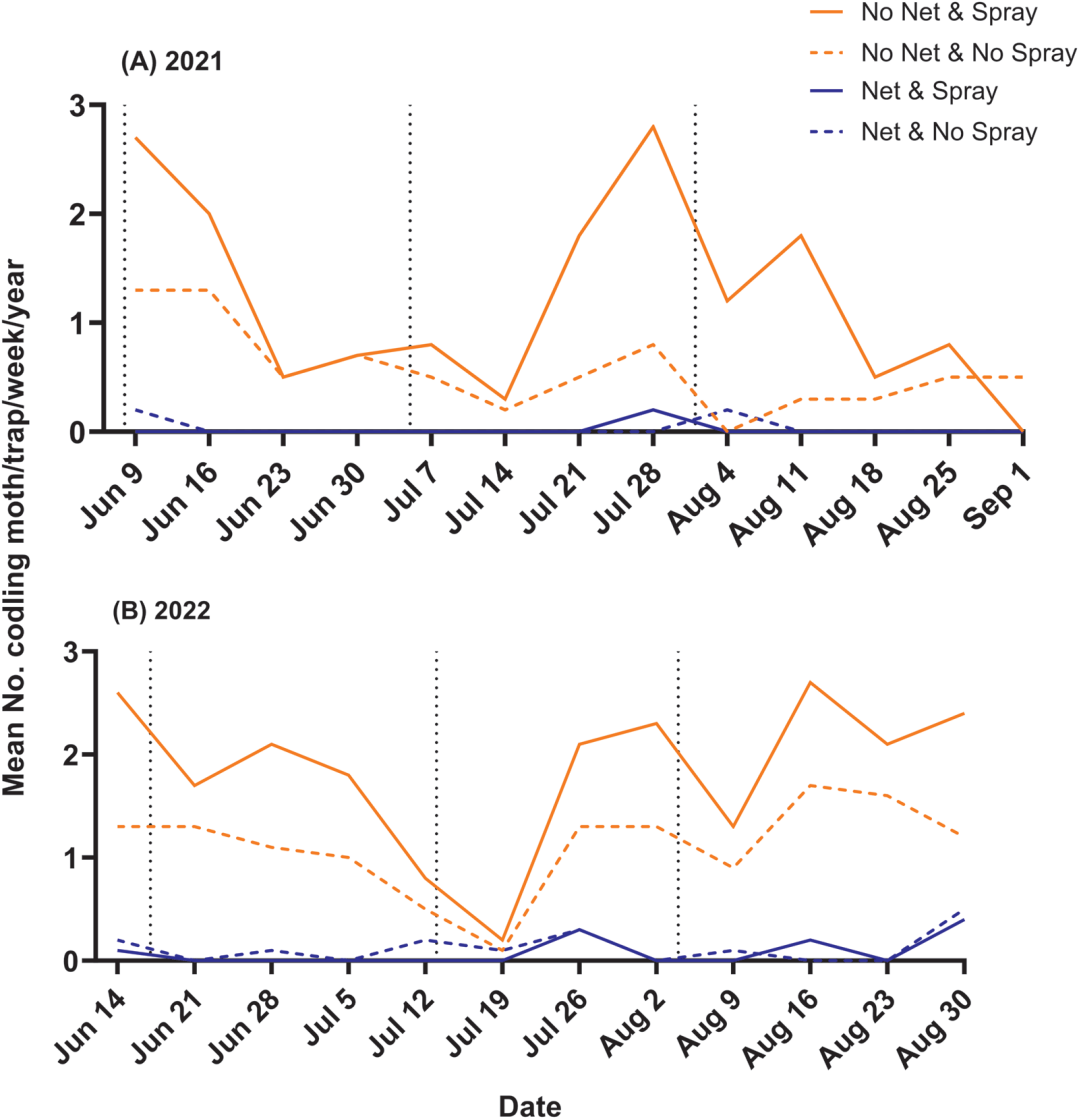
Trap catch results for the codling moth, combined for both White Bear Lake and Preston locations. Weekly mean trap catch across all treatments in 2021 (A), and 2022 (B). Dates for trapping in 2021 ranged from 9 June–1 September, and in 2022 from 14 June–1 September. Vertical dashed lines represent insecticide applications (see Table 2).

Codling moth adults were present in both orchards during 2021 and 2022. In the untreated check treatment (No Net & No Spray), they were captured in traps following a bivoltine emergence pattern (Fig. 3). In 2021, the first generation of codling moth had already emerged and begun to decline by the time traps were deployed (Fig. 3A). In 2022, the first generation of codling moth was on the decline at the time of the installation of our traps (Fig. 3B). Season-long averages for each experimental block under netting averaged less than 1.0 moth*/*trap for all blocks, with 3 out of 14 netted blocks catching 0 moths all season (Fig. 3A).

**Fig. 4.**
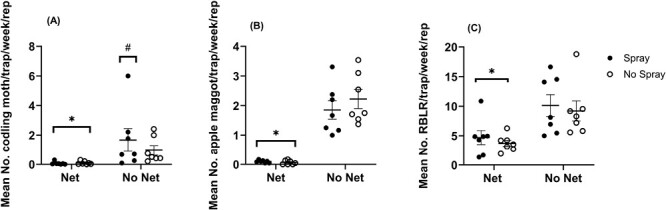
Average season-long trap catch per treatment for each of the 7 replications in both White Bear Lake and Preston over both years. Points depict season-long averages per replication for codling moth (A), apple maggot (B), and red-banded leafroller (C). There were 7 replications total, with 2 in 2021 and 5 in 2022. The horizontal line within each bracket depicts the mean across all replications, years, and locations, and the bracket represents the SEM of the 7 replications. *Treatments with significantly lower trap catch numbers compared to the untreated check treatment (No Net & No Spray). ^#^Treatments with significantly higher trap catch numbers compared to the untreated check treatment.

Trap catches differed in the presence of netting, insecticide application, and the interaction of these factors (Fig. 4A). The untreated check treatment resulted in a mean of 0.956 ± 0.332 (SE) codling moths*/*trap/week, compared to the netting treatment that reduced trap catch by a factor of ten to a mean of 0.097 ± 0.042 (SE) moths/trap/week (*F* = 218.52, df = 1, 723, *P* < 0.0001). The application of insecticide did not reduce codling moth trap catch, but instead, we observed significantly higher numbers caught in sprayed treatments with a mean of 1.667 ± 0.779 (SE) moths/trap/week ( *F* =9.67, df = 1, 723, *P* = 0.0019). The spray and net treatment had the lowest number of codling moths caught, with a mean of 0.063 ± 0.038 (SE) per trap/week. An interaction between spray and net methods (*F* = 12.49, df = 1, 723, *P* = 0.0004) may have been driven by 1 experimental block in White Bear Lake in 2022 that had considerably higher trap catches than the others, with a season-long average of 6.0 moths/trap/year. The means for each treatment across both years were: Net & Spray, 0.063 ± 0.038 (SE); Net & No Spray, 0.097 ± 0.042 (SE); No Net & Spray, 1.667 ± 0.779 (SE); and No Net & No Spray, 0.956 ± 0.332 (SE) (Fig. 4A). Averaged across experimental blocks and the 2 spray treatments, codling moth trap catch under netting was 0.08/trap/week; and without netting, was 1.31 trap/week. Based on these averages, the number of codling moth was reduced by 94% under the netting.

#### Apple maggot.

**Fig. 5.**
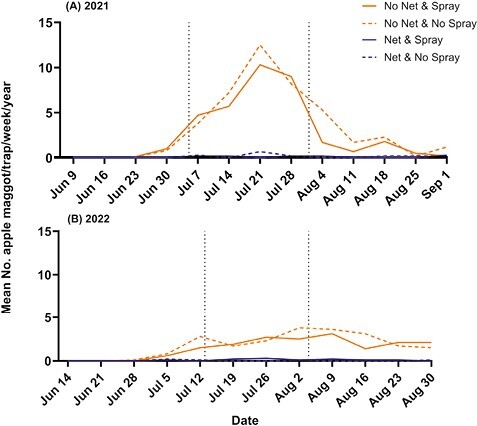
Trap catch results for the apple maggot, combined for both White Bear Lake and Preston locations. Weekly mean trap catch across all treatments in 2021 (A), and 2022 (B). Dates for trapping in 2021 ranged from 9 June to 1 September and in 2022 from 14 June to 1 September. Vertical dashed lines represent insecticide applications (see Table 2).

Apple maggot adults were present in both orchards in both 2021 and 2022. In the untreated check treatment, they were captured in traps following a univoltine emergence pattern (Fig. 5). In 2021, the first trap catch was recorded on 30 June (Fig. 5A). In 2022, the first trap catch was recorded on 28 June (Fig. 5B). Season-long averages for each experimental block under netting were all less than 0.2 flies/trap/week, with 3 blocks in 2022 catching 0 flies all season (Fig. 4B).

Traps within the untreated check treatment averaged a catch of 2.222 ± 0.295 (SE) flies/trap/week. By comparison, traps under the netting averaged 0.062 ± 0.024 (SE) flies/trap/week, a significant reduction in apple maggot trap catch (*F* =384.40, df = 1, 723, *P* < 0.0001). Insecticide use did not affect the number of apple maggot (*F* = 1.60, df = 1, 734, *P* = 0.2058). The net and insecticide interaction treatment did not significantly influence apple maggot trap catch numbers (*F* = 2.03, df = 1, 723, *P* = 0.1551). The means for each treatment across both years were: Net & Spray, 0.094 ± 0.016 (SE); Net & No Spray, 0.062 ± 0.024 (SE); No Net & Spray, 1.852 ± 0.289 (SE); and No Net & No Spray, 2.222 ± 0.295 (SE) (Fig. 4B). Averaged across experimental block s and the 2 spray treatments, apple maggot trap catch under netting was 0.08 flies/trap/week; and without netting, was 2.04 flies/trap/week. Based on these averages, the number of apple maggot was reduced by 96% under the netting.

#### Red-banded leafroller.

**Fig. 6.**
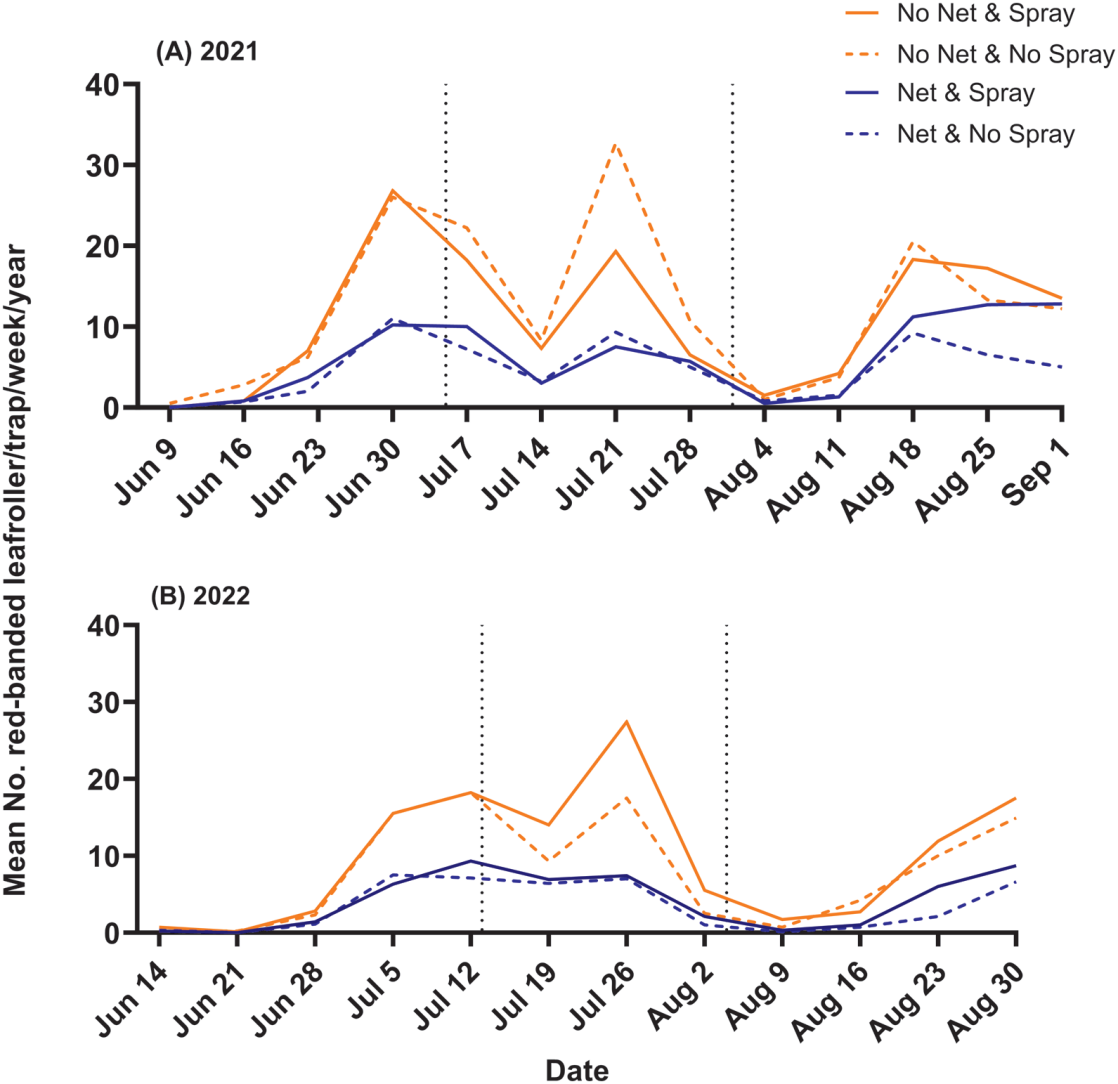
Trap catch results for the red-banded leafroller, combined for both White Bear Lake and Preston locations. Weekly mean trap catch across all treatments in 2021 (A), and 2022 (B), Dates for trapping in 2021 ranged from 9 June–1 September, and in 2022 from 14 June to 1 September. Vertical dashed lines represent insecticide applications (see Table 2).

Red-banded leafroller (RBLR) adults were present in both orchards in both 2021 and 2022. In the untreated check treatment, RBLR were captured in traps following a multivoltine emergence pattern consisting of 3 adult generations (Fig. 6). In both 2021 and 2022, we observed higher trap catches for this species compared to codling moth and apple maggot (Fig. 6). Traps in the untreated check treatment caught a mean of 9.201 ± 0.64 (SE) RBLR/trap/week. By comparison, the presence of netting significantly decreased trap catch numbers to an average of 3.723 ± 0.53 (SE) moths/trap/week (*F* = 189.41, df = 1, 723, *P* < 0.0001). Insecticide use did not influence the number of red-banded leafrollers caught (*F* = 3.55, df = 1, 723, *P* = 0.0601). The interaction of net and spray did not influence trap catch numbers (*F* = 0.94, df = 1, 723, *P* = 0.3334). The means for each treatment across both years were: Net & Spray, 4.696 ± 1.18 (SE); Net & No Spray, 3.723 ± 0.53 (SE); No Net & Spray, 10.115 ± 1.82 (SE); and No Net & No Spray, 9.201 ± 0.64 (SE) (Fig. 4C). The overall mean trap catch of red-banded leafroller across all experimental block s and dates was 4.209 ± 0.486 (SE) under the netting and 9.658 ± 0.457 without netting. Based on these means, the number of red-banded leafroller was reduced by 56% under the netting.

### Apple Quality

**Fig. 7.**
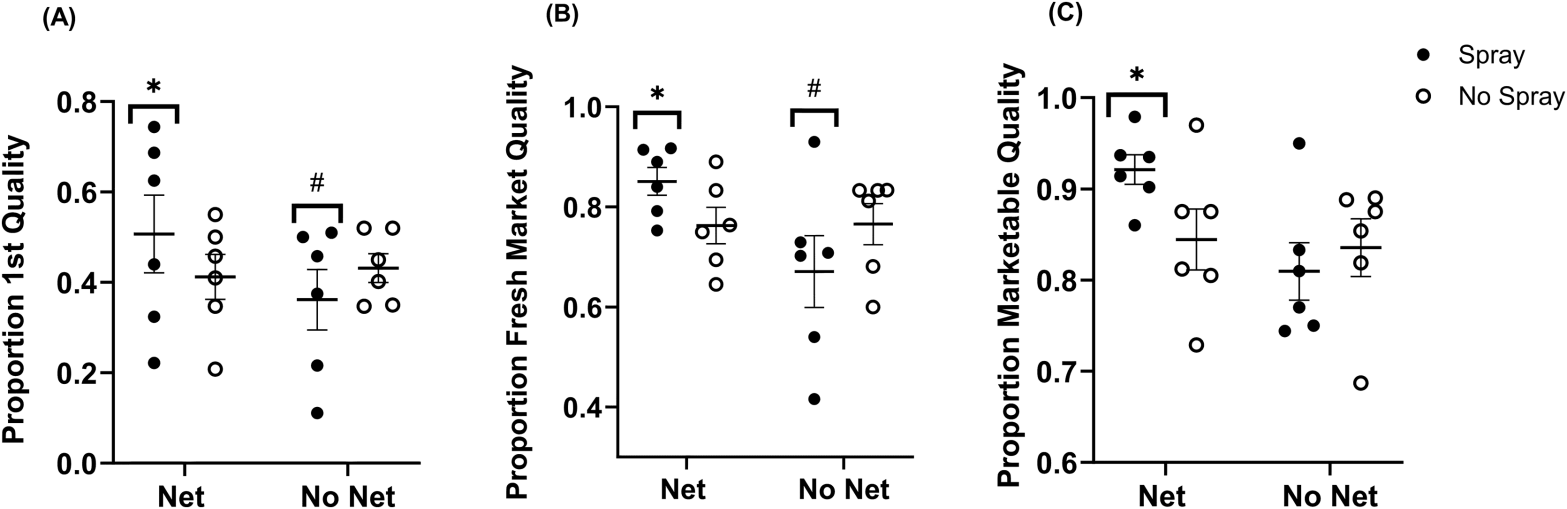
Proportions of quality ratings in 3 separate analyses. 1st Quality (A), Fresh Market Quality (B), and Marketable Quality (C). Proportions of fruit within each category are graphed for 6 replications between 2021 and 2022, with 2 in 2021 and 4 in 2022.The horizontal dash depicts the mean across all replications across both years, and the bracket represents the SEM. *Treatments with significantly higher proportions compared to the untreated check treatment (No Net & No Spray). ^#^Treatments with significantly lower proportions compared to the untreated check treatment.

In 2021 and 2022, we collected data from 1,350 apples and recorded their quality based on several criteria (see Materials and Methods, Supplementary Table 1). We assessed 678 apples taken from White Bear Lake, MN, and 672 taken from Preston, MN over 2 yr. The first analysis assessed the effect of our treatments on the fruit quality, and whether they influenced the ratio of apples within 2 categories: 1st quality apples (EF or F) vs. apples that were not (Fig. 7A). In total, 548 apples were of 1st quality compared to 802 apples that were not. The untreated check (no net or insecticide treatments) had a 44.3% likelihood of apples being of 1st quality. The spray treatment had a 34.4% likelihood of 1st quality fruit (*Z* = −2.54, df = 1342, *P* = 0.0113). The use of netting did not significantly change the proportion of fruit that were 1st quality (*Z* = −0.85, df = 1342, *P* = 0.3932). Combining the net and spray treatments synergized the likelihood of an apple being 1st quality to 62.5% (*Z* = 3.26, df = 1342, *P* = 0.0011).

The second analysis assessed the ratio of apples that were able to be sold fresh within each of our treatments (Fig. 7B). In total, there were 1,015 apples that were of fresh market quality and 335 that were not. The untreated check treatment had a 76.7% likelihood of fresh market quality fruit. The spray treatment significantly lowered the likelihood of fresh market quality fruit to 64.5% (*Z* = −3.45, df = 1342, *P* = 0.0006). The net treatment did not significantly influence the proportion of fresh market fruit (*Z* = −0.20, df = 1342, *P* = 0.8405). Combining spray and net treatments synergized the proportion of fresh market quality fruit to 90.9% (*Z* = 4.25, df = 1342, *P* < 0.0001).

The third analysis assessed the ratio of apples that were able to be marketed at all, whether sold fresh market or used in processing, to the apples that were unusable (cull). In total, 1,154 apples were of marketable quality and 196 apples were unmarketable (Fig. 7C). The untreated check treatment had an 83.1% likelihood of fruit being marketable. The net treatment did not significantly influence the proportion of marketable fruit (*Z* = 0.20, df = 1342, *P* = 8402). The net treatment did not significantly influence the proportion of marketable fruit (*Z* = −1.02, df = 1342, *P* = 0.3079). Utilizing both the net and spray treatments resulted in a significantly higher likelihood of marketable fruit at 93.1% (*Z* = 3.13, df = 1342, *P* = 0.0018).

The analyses of individual parameters of fruit quality were analyzed to determine if insect control methods (spray and net) influenced fruit production. Fruit size, russeting, and deformities were not influenced by net, spray, or their interaction, but did differ between years and/or experimental blocks. Red skin color was significantly lower in the netted treatment compared to un-netted (*Z* = −2.01, df = 1343, *P* = 0.0444). Skin color was not significantly influenced by the spray treatment (*Z* = −1.71, df = 1343, *P* = 0.0877) or the interaction of the spray and net treatments (*Z* = 0.87, df = 1343, *P* = 0.3831). The analysis of soluble solids (brix) revealed that most of the variability in fruit ripeness was driven by differences among experimental blocks and years. The netting treatment was found to have a significantly higher percentage of soluble solids (*F* = 5.73, df = 1, 47, *P* = 0.0208) while the spray treatment did not significantly influence soluble solid content (*F* = 0.015, df = 1, 47, *P* = 0.9003). The interaction of the net and spray treatments significantly decreased the percentage of soluble solids (*F* = 6.35, df = 1, 47, *P* = 0.0152).

### Apple Yield

**Fig. 8.**
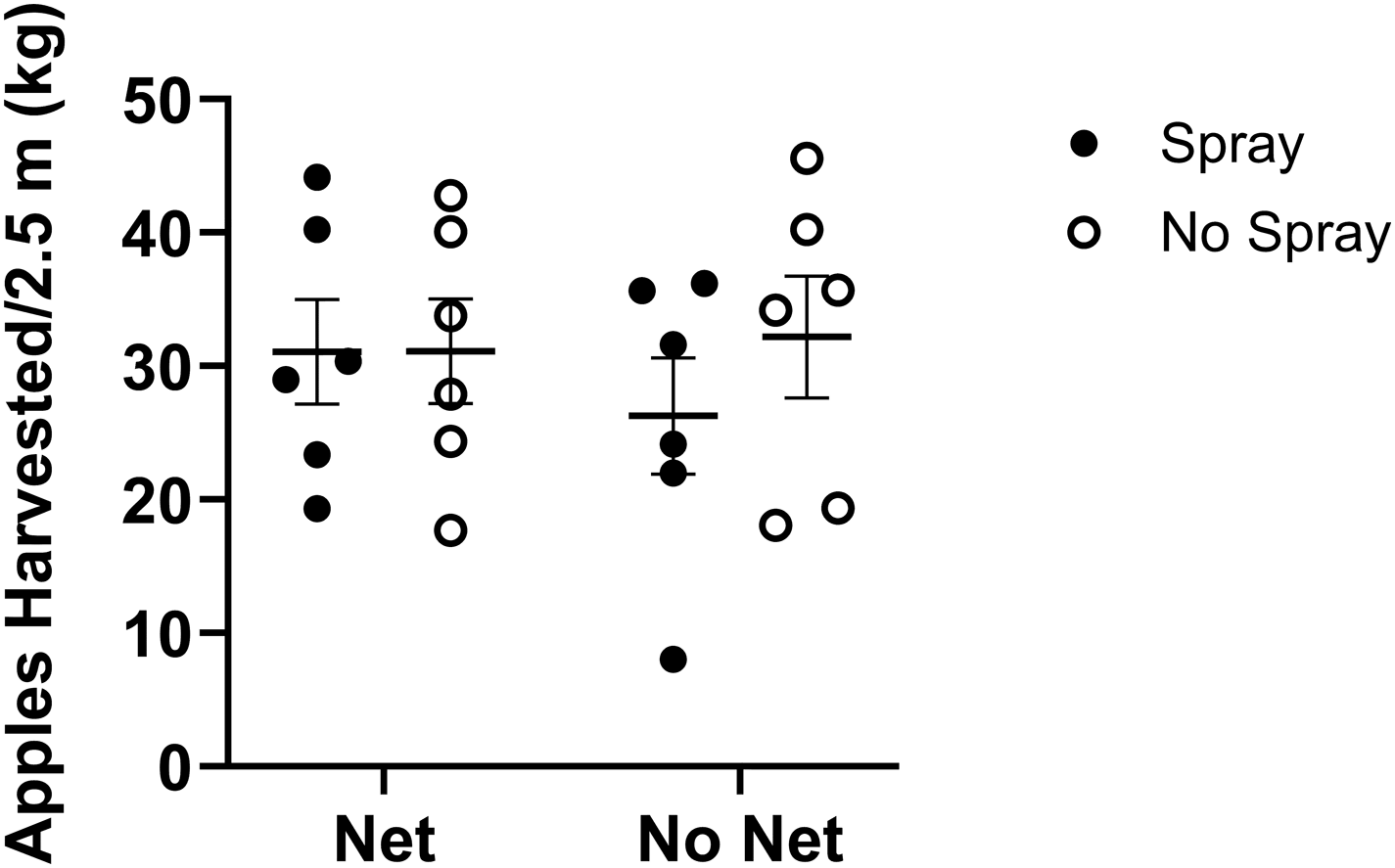
Yield data from each replication in 2021 and 2022. Yield (kg) graphed for 6 replications between 2021 and 2022, with 2 in 2021 and 4 in 2022. The horizontal dash depicts the mean across all replications across both years in each treatment, and the bracket represents the SEM. No significant differences were found between treatments.

Yield was not influenced by net, spray, or their interaction (*P* > 0.05) (Fig. 8). The mean for the untreated check treatment was 32.269 ± 4.25 (SE) kg/2.5 m row harvested. These findings are consistent with observations in the field during harvest; there was little variability in fruit-bearing on trees in adjacent rows within each experimental block that were under different experimental treatments.

### DAT and DLI

The mean daily temperatures were not significantly influenced by the presence of netting in either year (*P* > 0.05) (Table 3). The daily average temperature across both years was 24.02 °C in rows not covered in netting and 23.94 °C in rows covered in netting. Results from the ANOVA indicated no significant difference between treatments (*F* = 0.11, df = 1, 605 *P* = 0.7761). There were no statistical analyses of DLI data because only 2 PAR loggers were used in each year; DLI values are reported in Table 3.

**Table 3.** Mean daily temperature and daily light integral, ± SEM, recorded during 2021 and 2022 at White Bear Lake and Preston, MN. Photosynthetic active radiation (PAR) loggers were only placed at White Bear Lake in both years.

Year	Location	Net	Mean Daily Temperature (˚C)	Mean Daily Light Integral
2021	White Bear Lake	No	24.61 ± 0.70	437.73 ± 21.60
		Yes	24.54 ± 0.66	1,188.67 ± 46.44
	Preston	No	22.94 ± 0.57	—
		Yes	23.40 ± 0.59	—
2022	White Bear Lake	No	23.55 ± 0.55	1,234.06 ± 52.06
		Yes	23.46 ± 0.54	1,263.18 ± 39.01
	Preston	No	24.79 ± 0.54	—
		Yes	24.14 ± 0.54	—

Values shown are averages for the season. Daily Light Integral calculated with PAR data.

## Discussion

The results of this study documented hail netting as an effective method of control for 3 species of insect pests in Minnesota apple, particularly in cases of low to moderate insect pressure. In both 2021 and 2022, hail netting (1.8 × 6 mm mesh) significantly reduced the trap catch of all 3 insect species: codling moth was reduced by 94%, apple maggot by 96%, and the red-banded leafroller by 56%. These results indicate that netting alone may be able to control for a complex of pests without additional control measures, depending on insect pressure. For codling moth, we observed low pest pressure in both 2021 and 2022, as the average trap catch was never higher than the established action threshold of 5 moths/trap/week (Gleason et al. 1994, Beckerman et al. 2021). For apple maggot, the action threshold is 5 flies/trap/week in Minnesota (Stanley et al. 1987, Beckerman et al. 2021). We observed moderate pest pressure in 2021, with trap catches peaking above 10 flies/trap/week in our un-netted treatments. In 2022, apple maggot pressure was low, and never rose above the action threshold. There were individual traps at both locations over the 2 yr that exceeded the action threshold for codling moth or apple maggot and thus warranted an insecticide application from the grower. Despite observing traps that reached the action thresholds, we found very little fruit damage caused by insects in our experimental blocks. In apple, like other fruit crops, the development of an action threshold is based on empirical studies of pest phenology and fruit damage rather than a clearly defined economic injury level (Whalon and Croft 1984). These action thresholds, while widely accepted, could be adjusted for an orchard using hail netting as they may be more conservative than necessary to maintain low levels of fruit damage in an orchard using netting. There is no action threshold established for the red-banded leafroller, and while we observed higher trap catches of this species compared to the other species, we observed little-to-no leafroller damage, including surface feeding damage, in our experimental blocks.

Our findings are consistent with previous exclusion netting studies in apple. The use of single row netting has been documented to exclude the apple pest complex in several different climates and continents (Sauphanor et al. 2012, Alaphilippe et al. 2016, Chouinard et al. 2019). Most recently, Marshall and Beers (2021) found that netting (2 × 5 mm mesh) in Washington State also provided effective exclusion of codling moth. In their study, they used cage frames to support the netting, to accommodate a conventional round-tree experimental orchard. Subsequently, Marshall and Beers (2023) concluded that single row netting would likely be advantageous over large cage systems, in that it is more difficult for codling moth to colonize, mate, and develop a sizeable population within the single row trellis system. Additionally, it has been documented in this study and others that netting manufactured for its anti-hail properties is an effective exclusion tool for insect pests (Baiamonte et al. 2016). Netting could also cause an interruption in host-seeking behavior in the apple maggot, as they seek out trees and fruit primarily using visual and olfactory cues, but we are unaware of experiments testing this in the field (Moericke et al. 1975, Prokopy and Roitberg 1984, Forbes and Feder 2006). The use of white Drape Net hail netting, the same used in this study, was recently documented as an effective method for codling moth exclusion and sunburn prevention in New York State (Aćimović and Jentsch 2020). Hail netting has the potential to be an important nonchemical control option in Minnesota and other North American apple regions, giving growers an additional practical strategy toward developing more sustainable IPM programs.

For all pests, there were no significant increases in pest suppression with the application of insecticides. Somewhat surprisingly, we found that insecticide application significantly increased the trap catch of codling moth. However, this may have been driven by high season-long trap catches in a single experimental block in the White Bear Lake orchard in 2022, where there was a strong interior edge effect (Knight 2007). For the apple maggot, it is possible that the active ingredient used to target this pest, acetamiprid (Assail), did not offer suppression levels that we could detect in our study. We believe insecticide drift between experimental rows to be minimal, as all insecticides were applied in the early morning at low wind speeds and moderate temperatures. Regardless, the netting was able to produce significant changes in pest suppression, while the insecticide treatment did not.

It has been observed in previous studies that there is potential for outbreaks of secondary pests such as the European red mite, aphids, and leafrollers under netting systems (Marshall and Beers 2021, 2022, Chouinard et al. 2022). While we did not observe any secondary pest infestations in this study, or any fruit damage or physiological symptoms of such pests under the netting, additional investigation into colonization under hail netting in Minnesota is needed. A prolific invasive species, the brown marmorated-stink bug (BMSB, *Halyomorpha halys)*, has also been documented as a potential threat to cold-hardy varieties in Minnesota orchards (Shanovich et al. 2019). In Italy and Washington State, the use of exclusion netting has already been shown to be efficacious against this pest (Candian et al. 2018, Marshall and Beers 2022). The continued investigation into hail netting’s influence on secondary and invasive pests will help to inform practical IPM implementation for more sustainable apple production systems in Minnesota and the Midwest region.

The use of hail netting supplemented with insecticide application can protect fruit from insect pest damage and increase the proportion of first-quality, fresh market, and generally marketable fruit in a high-density production system. We found that the Net & Spray treatment had significantly higher proportions of desirable fruit compared to our untreated check (No Net & No Spray) treatment in all 3 of our analyses: the likelihood of 1st-quality fruit improved from 47 to 62%, the likelihood of fresh market fruit improved from 76.7 to 90.9%, and the likelihood of producing marketable fruit improved from 83.1 to 93%. Importantly, while netting alone did not result in a significant improvement in the proportion of marketable fruit, it also did not result in any negative changes. Conversely, the use of insecticide alone was found to have significantly lower proportions of 1st quality and fresh market quality fruit. This result indicates that insecticide alone did not provide the same protection from damage that the netted treatments achieved.

Our findings, using SweeTango, are consistent with previous studies that have examined effects of netting on fruit production. A study examining the influence of Drape Net on apple yield and quality in New York found that there are variable results dependent upon the cultivar (Aćimović and Leffelman 2021). For instance, Honeycrisp and other varieties experienced no negative side effects from the netting, while others had decreased yield, sugar accumulation, and/or color development (Aćimović and Leffelman 2021). A study using hail netting in Brazil found that there were no changes in color or fruit quality in fruit grown under netting, but there was a short delay in fruit maturation (Bosco et al. 2015). In many studies, results have varied amongst cultivars, growing conditions, climate, and location, as all of these factors interact with netting and largely impact fruit production (Manja and Aoun 2019). We also observed no significant changes in apple yield among experimental treatments. Variation within our yield data was driven by differences between our experimental blocks and differences between years. These findings were consistent with observations that were made in the field during harvest procedures. Overall, our findings indicate that hail netting has no major negative impacts on fruit quality or yield, and can support decreased use of conventional insecticides on the production of SweeTango apples in Minnesota.

Hail netting presents a promising solution for growers in search of nonchemical control alternatives, either to minimize environmental impact or address the declining efficacy of chemical control methods. While this netting can last many years, and does not cause any chemical drift and runoff into the environment, it is not a perfect solution in terms of environmental impact. This netting is constructed with monofilament, or nylon, a petroleum-based product. This makes responsible disposal of the netting at the end of its lifetime a challenge. Recent developments in bio-polymer nets, which have less developmental costs and do not rely on petroleum products, may provide for a greener option in the future of exclusion netting for sustainable IPM (Chouinard et al. 2022). The findings of our study could be used to inform grower practices and IPM tactics for pest suppression in Minnesota and Midwest apple production. The use of hail netting, in addition to hail protection, has potential to offer significant reductions in pest pressure and significant protection to high-value varieties of apples. Scouting for insects inside the netting, and supplementing with insecticide application only as needed, will continue to minimize risk to fruit production and potentially reduce the chemical inputs in the production system. As we continue to seek sustainable IPM practices in Midwest apple production, future research should be conducted to understand the influence of netting on insect feeding and mating behavior, if there are changes in efficacy at high pest pressure, and whether insects can adapt over time to this netting system. Finally, more research is needed to assess the potential nontarget risks of netting to natural enemies and biocontrol services from generalist predators and parasitoids in Midwest apple production systems.

## Supplementary Material

toad197_suppl_Supplementary_Tables_1
