# Supplementary material for: Hail netting excludes key insect pests and protects from fruit damage in a commercial Minnesota apple orchard
Source: J Econ Entomol. 2023 Oct 31;116(6):2104–15. doi: 10.1093/jee/toad197 (PMC10711545; doi:10.1093/jee/toad197)
Supplement: toad197_suppl_Supplementary_Tables_1 [file toad197_suppl_supplementary_tables_1.docx]

***Supplementary Table 1***: Parameters for fruit rating and rating scale used for SweeTango™ apples.

| Parameter | Description |  | Rating |
| --- | --- | --- | --- |
| Red Skin | % skin surface red in color | >80% | EF |
|  |  | 60-79% | F |
|  |  | 20-60% | M |
|  |  | <20% | U |
| Size | Diameter of apple (mm) | >69.5 | EF/F |
|  |  | 63.5-69.5 | M |
|  |  | <63.5 | U |
| Russeting | % surface webbed/blotched | <10% | EF |
|  |  | 10-25% | F |
|  |  | >25% | M |
| Deformity | Asymmetry/misshapenness | Present | U |
| Blemishes | Open wound/rotting | Present | C |
|  |  | Absent | EF/F/M/U |
|  | Closed wound (>9.5 mm) | Present | M/U |
|  |  | Absent | EF/F |
|  | Closed wound (<9.5 mm) | Present | F/M/U |
|  |  | Absent | EF |
